# PKM2 under hypoxic environment causes resistance to mTOR inhibitor in human castration resistant prostate cancer

**DOI:** 10.18632/oncotarget.25498

**Published:** 2018-06-12

**Authors:** Yota Yasumizu, Hiroshi Hongo, Takeo Kosaka, Shuji Mikami, Koshiro Nishimoto, Eiji Kikuchi, Mototsugu Oya

**Affiliations:** ^1^ Department of Urology, Keio University School of Medicine, Tokyo, Japan; ^2^ Division of Diagnostic Pathology, Keio University School of Medicine, Tokyo, Japan; ^3^ Department of Uro-Oncology, Saitama Medical University International Medical Center, Hidaka, Japan

**Keywords:** CRPC, PKM2, PI3K-Akt-mTOR signaling pathways, hypoxias, C4-2AT6

## Abstract

The aim of this study was to explore the efficacy of mTOR inhibitor for castration-resistant prostate cancer (CRPC) under hypoxia. Although under normoxia C4-2AT6, it is a CRPC cell line, expressed elevated pAkt, pS6 and Pyruvate kinase M2 (PKM2) accompanied by elevated HIF-1a expression, 5% hypoxic condition further induced expression of these proteins. These results indicate hypoxic environment elevated PI3K/Akt/mTOR pathway in aggressive prostate cancer. However, C4-2AT6 cells treated with mTOR inhibitor under hypoxia less decreased compared to cells treated with the same dose drugs under normoxia. Western blot analysis showed mTOR inhibitor: RAD001 not only inhibited pS6, but also increased the expression of PKM2 in a dose and time dependent manner. Pyruvate kinase acts on glycolysis. PKM2, which is frequently express in tumor cells, is one isoform of pyruvate kinase. PKM2 is reported to act as a transcription factor. In the present study overexpression of PKM2 in C4-2AT6 induced resistance to RAD001 under normoxia. To evaluate the therapeutic effect of targeting PKM2, we inhibited PKM2 in C4-2AT6 under hypoxia using si-PKM2. The number of C4-2AT6 under chronic hypoxia exposed to siPKM2 significantly decreased compared to intact C4-2AT6 under chronic hypoxia. Furthermore, si-PKM2 improved resistance to mTOR inhibitor in C4-2AT6. When examined using clinical samples, high PKM2 expression was correlated with a high Gleason score and poor PSA free survival. These results suggested that up-regulation of PKM2 is one possibility of resistance to mTOR inhibitor in CRPC. And it is possible that PKM2 is a useful therapeutic target of CRPC.

## INTRODUCTION

Prostate cancer is the most frequently diagnosed cancer and the second leading cause of cancer related death in the United States [1, 2]. Most metastatic prostate cancer acquire androgen independent growth ability and show resistance to androgen deprivation therapy (ADT) [3]. This is so-called castration-resistant prostate cancer (CRPC). In addition to docetaxel, recently other novel drugs have shown promising results in prolonging survival, including the cytochrome P17 inhibitor abiraterone acetate, the anti-androgen enzalutamide, cytotoxic drug cabazitaxel, and the bone-targeting radiopharmaceutical radium-223 dichloride leading to approval in Japan [4, 5, 6, 7, 8]. However, the anti-tumor effect of these drugs is limited. Therefore, the further investigation about CRPC is needed to establish a new treatment strategy for CRPC.

Hypoxia is an essential feature of the microenvironment of solid tumors. As with other solid tumors, prostate cancer has the characteristic of hypoxia. Assessment of intratumoral oxygen levels by a polarographic needle oxygen electrode identified that the oxygen tension (pO2) in prostate cancer tissue was significantly lower than in normal tissue [9]. To understand characteristics of prostate cancer, we need to elucidate microenvironmental regulation under hypoxia in CRPC. Energy production in tumor cells exposed to hypoxia shifts from oxidative phosphorylation through a mitochondria to glycolysis. It is called Warburg effect. This metabolic reprogramming activates an anabolic metabolism, and contributes to the establishment of intracellular environment which was suitable for proliferation to cancer cells [10]. Pyruvate kinase catalyzes the last step of glycolysis, transferring the phosphate from phosphoenolpyruvate (PEP) to ADT to yield ATP and pyruvate. Normal cells generally express the pyruvate kinase M1 (PKM1) isoform, whereas tumor cells frequently switch to PKM2 expression [11]. PKM2, a key protein of Warburg effect, shows low activity of the enzyme in contrast to PKM1 which has high constitutive activity [12]. Low PKM2 activity is believed to provide growth advantage for tumor progression as it helps to change the carbon source from glycolysis to biosynthesis [11, 13]. Converting PKM2 into PKM1, as well as PKM2 activators, suppress tumorigenesis [14].

We previously reported that long-term androgen ablation activated PI3K/Akt/mTOR signaling pathway, which explained in part the aggressiveness of CRPC [15], and that inhibition of PI3K/Akt/mTOR pathway showed anti-tumor effects on docetaxel resistant CRPC *in vivo* and *in vitro* [16]. However, the clinical trial of mTOR inhibitor in men with castrate-resistant prostate cancer has been disappointing with few responses and a short time to progression [17, 18]. We proposed a hypothesis that hypoxia and/or PKM2 is partly responsible for resistance of CRPC to mTOR inhibitor. In the present study, we intended to characterize microenvironmental regulation of PI3K/Akt/mTOR signaling pathways in hypoxic conditions and to elucidate the clinical value of PKM2 as a prognostic indicator.

## RESULTS

### PI3K/Akt/mTOR pathway is up-regulated in CRPC under hypoxia

**Figure 1:**
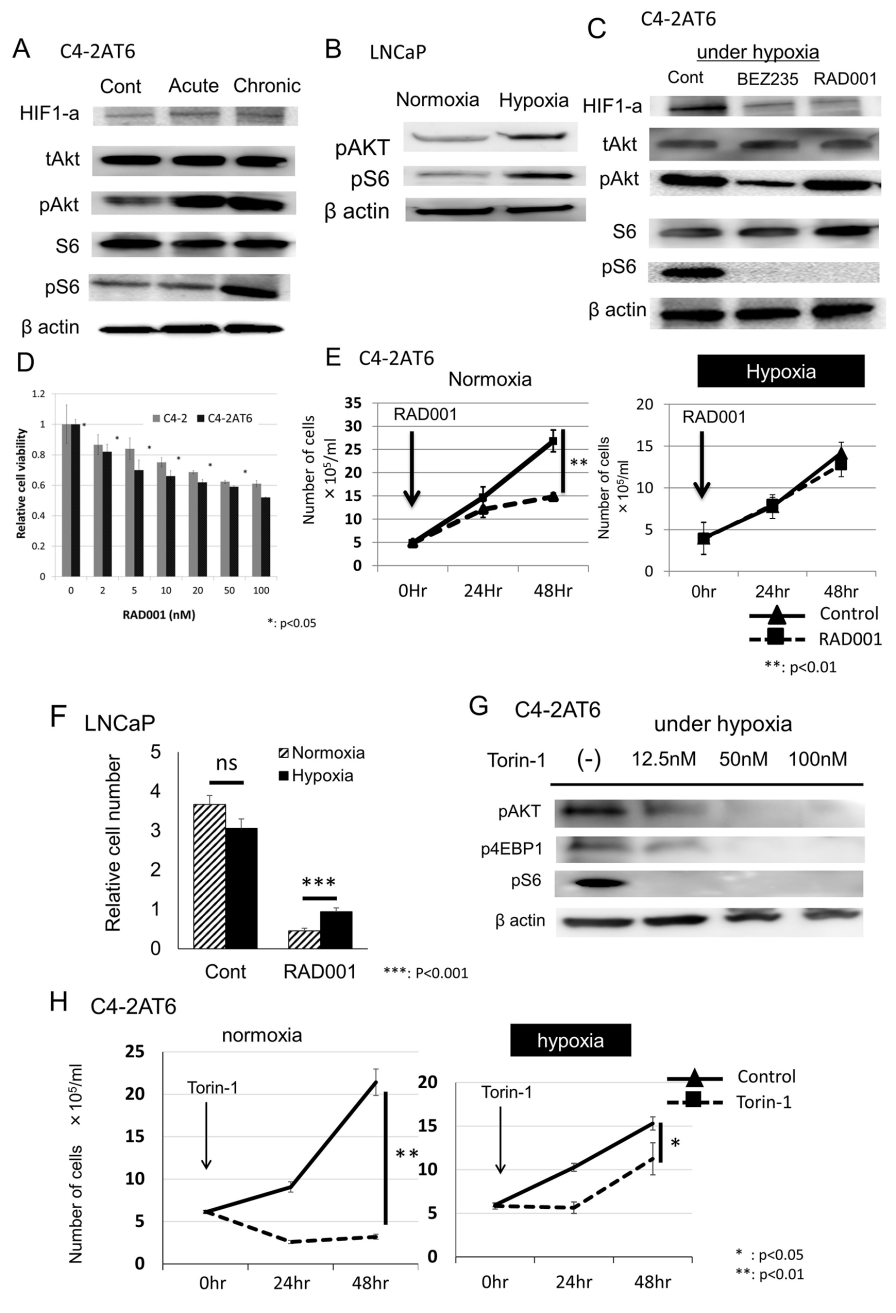
Hypoxia induces resistance to mTOR inhibitors in prostate cancer cells **(A)** Expression of HIF1-a, pAkt, pS6 in C4-2AT6 cells under normoxia, acute hypoxia, chronic hypoxia. “Acute” indicates cells cultured under hypoxia in one day, and “Chronic” cells cultured under hypoxia over one month. Expression of pAkt, pS6 under chronic hypoxia was up-regulated. **(B)** LNCaP under chronic hypoxia also showed higher expression of pAkt and pS6 compared to under normoxia. **(C)** Inhibitory effects of exposure to NVP-BEZ235 (500 nM) or RAD001 (100nM) for 24 hours on HIF1-a, pAkt, and pS6 in C4-2AT6 cells. NVP-BEZ235 inhibited the expression of pAkt and pS6. RAD001 inhibited the expression of pS6. **(D)** C4-2 cells and C4-2AT6 cells under normoxia were treated with RAD001. Cell viability was measured by WST assay. C4-2AT6 cells showed greater sensitivity to NVP-BEZ235 than C4-2 cells. ^*^p< 0.05 compared to C4-2 at the same dose. **(E)** The number of C4-2AT6 cells under normoxia and hypoxia 24hr or 48hr after administration of RAD001. RAD001 exhibited low cytotoxicity towards C4-2AT6 under chronic hypoxia. ^**^ p<0.01 compared to intact C4-2AT6 cells. **(F)** The number of LNCaP cells under normoxia and hypoxia 48hr after administration of RAD001. ^***^p<0.001 compared to intact LNCaP cells. **(G)** Inhibitory effects of exposure to Torin-1 for 24 hours on pAkt, pS6 and p4EBP1 in C4-2AT6 cells. **(H)** The number of C4-2AT6 cells under normoxia and hypoxia 24hr or 48hr after administration of Torin-1.

We previously reported that C4-2AT6 cells, which have higher resistance to docetaxel than C4-2 cells, show up-regulation of pAkt and sensitivity to PI3K/Akt/mTOR inhibtor [15, 16]. To evaluate characteristics of C4-2AT6 under hypoxia, we cultured C4-2AT6 under 5% O2. We prepared two cells, C4-2AT6 under acute hypoxia and C4-2AT6 under chronic hypoxia. “Acute” indicates cells cultured under hypoxia in one day, and “Chronic” cells cultured under hypoxia over one month. The expression of pAkt and phosphorylated S6 ribosomal protein (pS6) was significantly up-regulated under chronic hypoxia compared to under normoxia or acute hypoxia (Figure 1A). Similarly LNCaP under hypoxia showed higher expression of pAkt and pS6 compared to under normoxia (Figure 1B). This result indicated that hypoxic environment up-regulated PI3K/Akt/mTOR pathway. Next, we used PI3K/Akt/mTOR pathway inhibitor to inhibit the up-regulation of this pathway in C4-2AT6 under hypoxia. RAD001 is mTOR inhibitor and NVP-BEZ235 is a dual PI3K and mTORC1/2 inhibitor. The concentrations of RAD001 and NVP-BEZ235 were set at 100nM and 500nM, respectively, because these concentrations fully inhibited the PI3K/Akt/mTOR pathway in C4-2AT6 under normoxia [16]. Both of these drugs fully inhibited the expression of pS6, and NVP-BEZ235 also inhibited pAkt (Figure 1C). In addition, both drugs decreased the expression of HIF1-a in C4-2AT6 (Figure 1C). This result is consistent with previous reports [19].

### C4-2AT6 shows resistance to mTOR inhibitor under hypoxia

We affirmed the cytotoxic effect of RAD001 in C4-2 and C4-2AT6 cells under normoxia *in vitro* by WST assay. As the same of previous reports [15], C4-2AT6 showed higher sensitivity to RAD001 than C4-2 (Figure 1D). Then, we evaluated the cytotoxic effect of RAD001 to C4-2AT6 under hypoxia using the method of direct cell count. RAD001 (100nM) showed significant cytotoxic effects in C4-2AT6 under normoxia (Figure 1E, left). On the other hand, RAD001 exhibited low cytotoxicity towards C4-2AT6 under chronic hypoxia (Figure 1E, right). Similarly, LNCaP under hypoxia showed resistance to RAD001 compared to under normoxia (Figure 1F). Although Torin-1 also inhibited the PI3K/Akt/mTOR pathway under hypoxia, Torin-1 showed low cytotoxicity towards C4-2AT6 under hypoxia compared to C4-2AT6 under normoxia like RAD001 (Figure 1G & 1H). These results indicated that under hypoxic environment C4-2AT6 was regulated by not only PI3K/Akt/mTOR pathway but also other pathway.

### mTOR inhibitor up-regulats PKM2 under hypoxia

**Figure 2:**
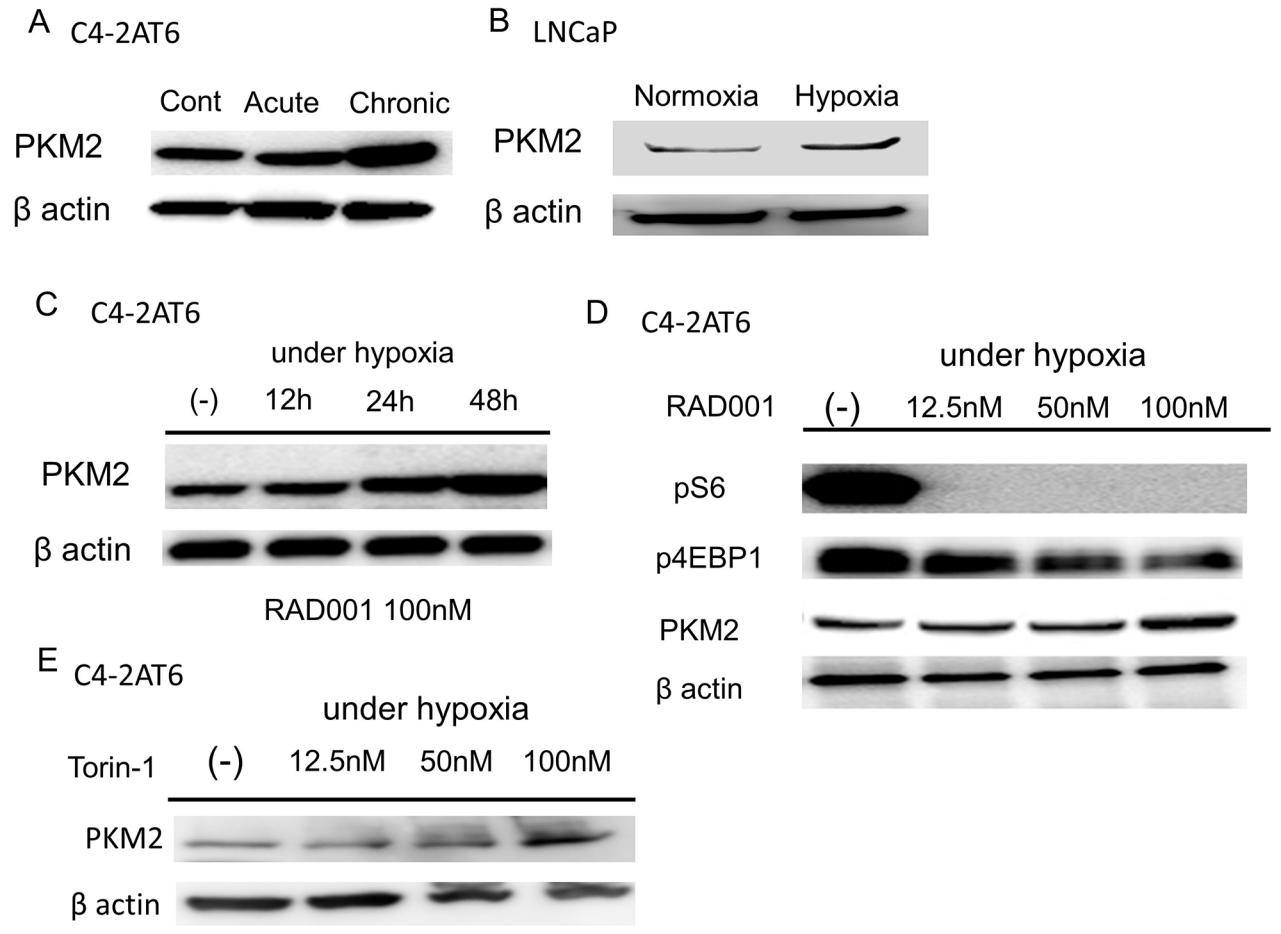
Hypoxia and mTOR inhibitor up-regulates the expression of PKM2 **(A)** Expression of PKM2 in C4-2AT6 cells under normoxia, acute hypoxia, chronic hypoxia. Expression of PKM2 under chronic hypoxia was up-regulated. **(B)** Expression of PKM2 under chronic hypoxia was also up-regulated in LNCaP. **(C)** The expression of PKM2 in C4-2AT6 under chronic hypoxia expose to 100nM RAD001 significantly increased in a time-dependent manner. **(D)** C4-2AT6 cells under chronic hypoxia were exposed for 24 hours to the concentrations of RAD001 indicated. pS6 and p4EBP1were inhibited by 50nM RAD001 and 100nM RAD001, respectively. The expression of PKM2 was up-regulated under 100nM RAD001. **(E)** Torin-1 also induced PKM2 expression in C4-2AT6 under hypoxia after 24 hours.

PKM2 exists in tumor cells instead of PKM1 and plays a key role in aerobic glycolysis [11]. Our hypothesis is that PKM2 regulates the resistance to PI3K/Akt/mTOR inhibitors in C4-2AT6 under hypoxia. The expression of PKM2 in C4-2AT6 under chronic hypoxia was significantly up-regulated compared to C4-2AT6 under normoxia or acute hypoxia (Figure 2A). PKM2 in C4-2AT6 was also up-regulated by exposure to 1% O_2_ (Supplementary Figure 1). In addition, long term treatment with low dose RAD001 also up-regulated the expression of PKM2 (Supplementary Figure 2). Similarly, PKM2 was elevated under hypoxia in LNCaP (Figure 2B). The expression of PKM2 in C4-2AT6 under chronic hypoxia expose to 100nM RAD001 significantly increased in a time-dependent manner (Figure 2C). In addition, the induction of PKM2 by 48h RAD001 under chronic hypoxia had dose-dependency (Figure 2D). Similar results were obtained under acute hypoxia (Supplementary Figure 3). Administration of 100nM Torin-1 increased the expression of PKM2 in C4-2AT6 (Figure 2E).

### PKM2 up-regulation facilitates mTOR resistance in CRPC cells

**Figure 3:**
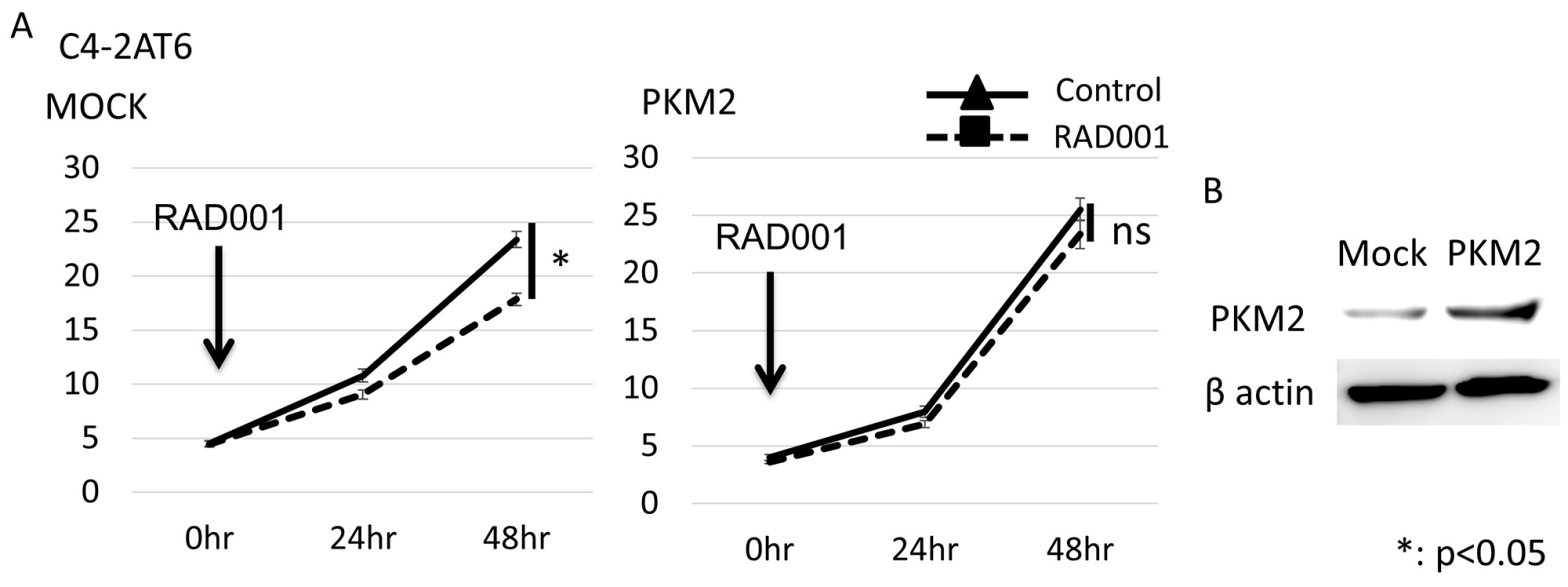
Overexpression of PKM2 in C4-2AT6 cells induces resistance to mTOR inhibitor under normoxia **(A)** The number of C4-2AT6 cells with overexpression of PKM2 under normoxia 24hr or 48hr after administration of RAD001. RAD001 exhibited low cytotoxicity towards C4-2AT6 with overexpression of PKM2 under normoxia. ^*^p< 0.05 compared to intact C4-2AT6 cells. **(B)** Western blotting showed PKM2 expressions was up-regulated in C4-2AT6 transfected with PKM2 plasmids.

We made C4-2AT6 with overexpression of PKM2 and examined whether overexpression of PKM2 induced resistance to a mTOR inhibitor under normoxia in C4-2AT6. The number of C4-2AT6 with overexpression of PKM2 was increased 48 hours after exposure compared to normal C4-2AT6. On the other hand, the number of C4-2AT6 with mock plasmid showed no remarkable change compared to normal C4-2AT6 (Figure 3A & 3B). These results suggested that PKM2, at least in a part, contributed to the resistance to the mTOR inhibitor in prostate cancer.

### Inhibition of PKM2 shows anti-tumor effect in CRPC cells

**Figure 4:**
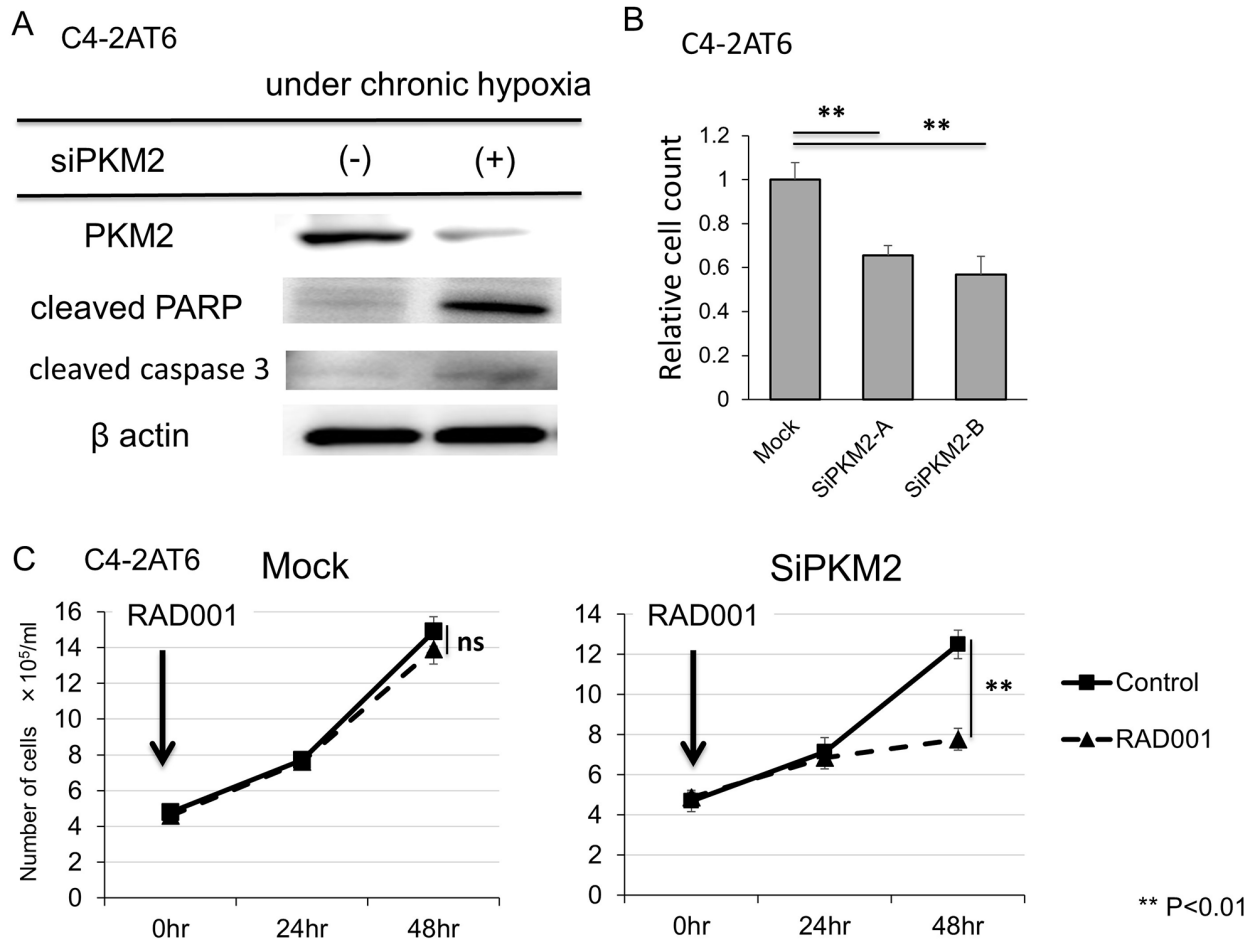
Inhibition of PKM2 shows anti-tumor effect and improves resistance to mTOR inhibitor in C4-2AT6 cells **(A)** The expression of PKM2 was inhibited and the expression of cleaved PARP and cleaved caspase3 was up-regulated in C4-2AT6 under chronic hypoxia exposed to si-PKM2 for 24 hr. **(B)** The number of C4-2AT6 under chronic hypoxia exposed to siPKM2 after 72 hours significantly decreased compared to intact C4-2AT6 under chronic hypoxia. **(C)** The number of C4-2AT6 cells under hypoxia exposed to both 100nM RAD001 and si-PKM2 for 48 hours significantly decreased compared to C4-2AT6 cells expose to RAD001 only, while the number of C4-2AT6 cells under hypoxia exposed to RAD001 and mock showed no remarkable difference with C4-2AT6 cells exposed to RAD001.

To evaluate the therapeutic effect of targeting PKM2, we inhibited PKM2 in C4-2AT6 under hypoxia using si-PKM2. Western blot analysis showed si-PKM2 significantly decreased the expression of PKM2 and up-regulated the expression of cleaved PARP. This result indicates the inhibition of PKM2 in C4-2AT6 under chronic hypoxia induced apoptosis (Figure 4A). Furthermore, in direct cell counting the number of C4-2AT6 under chronic hypoxia exposed to siPKM2 after 72 hours significantly decreased compared to intact C4-2AT6 under chronic hypoxia (Figure 4B). Next we examined significance of PKM2 expression for mTOR inhibitor sensitivity of CRPC. Direct cell counting showed PKM2 knockdown sensitized C4-2AT6 to RAD001 under chronic hypoxia (Figure 4C). These results suggested PKM2 induction by hypoxia modulates mTOR resistance in CRPC.

### Elevated expression of PKM2 correlates with biochemical recurrence of CRPC

**Figure 5:**
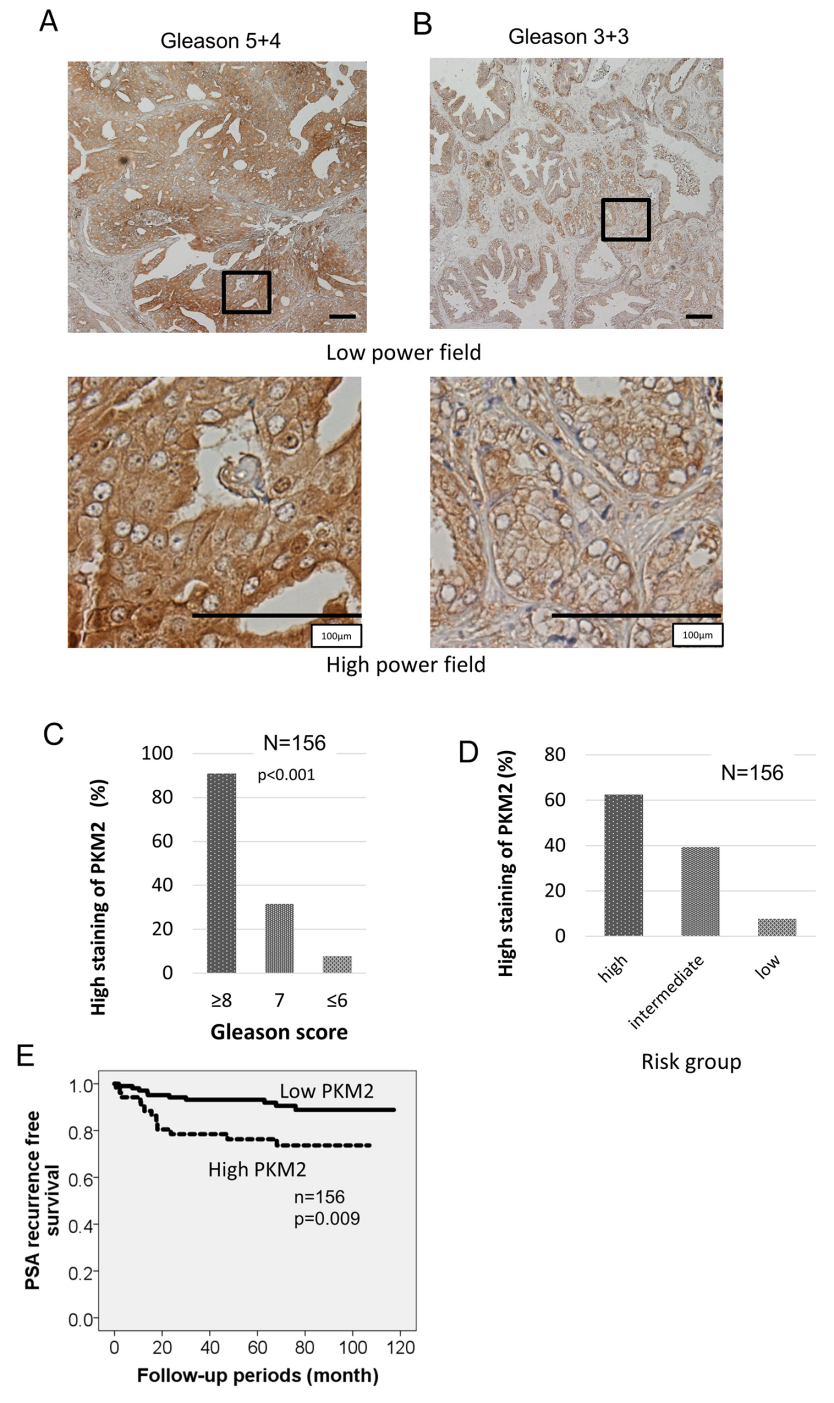
Clinical samples show elevated expression of PKM2 correlates with biochemical recurrence of prostate cancer after prostatectomy **(A, B)** Prostate cancer with the indicated Gleason grades was immunohistochemically stained with anti-PKM2 antibody. Representative images for each Gleason score were included. Scale bars represent 100 μm. **(C)** PKM2 was significantly up-regulated in high Gleason tissue. **(D)** Correlation between expression of PKM2 and risk group of prostate cancer. **(E)** Kaplan–Meier curves of PSA recurrence-free survival of the patients after surgery for prostate cancer according to the intensity of PKM2 was shown.

We evaluated the expression of PKM2 in human prostate cancer tissue using immunohistochemistry. Paraffin-embedded sections were obtained from 156 patients who underwent radical prostatectomy at Keio University Hospital. (Figure 5A & 5B). Two authors independently evaluated immunoreactivity. They assign 53 patients to “high intensity group” and the other 103 patients to “low intensity group”. Using chi-square test, PKM2 was significantly up-regulated in high Gleason tissue (Figure 5C). Next, we examined the trend of PKM2 expression in Low, Intermediate, and High risk prostate cancer. Patients with prostate cancer were assigned according to the NCCN guideline. As with the Gleason score of a specimen, the expression level of PKM2 was significantly different among high, intermediate and low risk groups in the chi-square test (Figure 5D, Table 1). We performed univariate analysis to determine the indicators for subsequent PSA recurrence following surgery. Kaplan-Meier analysis indicated strong expression of PKM2 was significantly correlated with the biochemical recurrence of prostate cancer, and the 5-year PSA recurrence-free survival rate was 95.4% in patients with high PKM2 expression compared to 78.8% in their counterparts (p<0.01, Figure 5E).

**Table 1:** Patients’ characteristics and PKM2 expression

		PKM2	
		Low	High
pre operative PSA	< 10	82	42
	10-20	18	10
	≥ 20	2	1
clinical stage	T1c, T2a	77	38
	T2b, T2c	25	11
	T3	0	3
Gleason score ^※^1	≤ 6	78	13
	7	20	32
	≥ 8	4	7
risk group	low	36	3
	intermediate	60	39
	high	6	10

^※^1 Gleason score at prostate biopsy.

## DISCUSSION

In the present study, we demonstrated the resistance of C4-2AT6 under hypoxia to PI3K/Akt/mTOR inhibitor, and the possibility of PKM2 involving to the resistance of mTOR inhibitor. In addition, we retrospectively evaluated the impact of PKM2 expression for PSA recurrence of immunohistochemical staining in a series of patients with prostate cancer. Our results suggested that high PKM2 expression was related to shorter PSA recurrence-free survival. Besides, in the microarray dataset from the GEO database (GSE16560), high expression of PKM2 was associated with significant decreases in overall survival in prostate cancer (p<0.001) (Supplementary Figure 4). To the best of our knowledge, this is the first study evaluating the prognostic value of PKM2 expression in patients with prostate cancer. It is important to understand the significance of PKM2 expression in clinical samples. In this study, we showed that PKM2 is one of the prognostic factors for PSA failure after prostatectomy. However, it is impossible to determine the connection between mTOR inhibitor resistance under hypoxia and PKM2 expression in clinical samples because mTOR inhibitors are not approved for CRPC treatment. This is a subject for future study.

PKM2 is an important regulator of cancer metabolism responsible for the Warburg effect. In most cancer cells, the conversion of PEP and ADP to pyruvate and ATP in the presence of oxygen generates the necessary amount of energy needed for rapid cellular proliferation. Several studies investigated that high expression of PKM2 is correlated with prognosis among several cancer patients such as breast cancer, hepatocellular carcinoma, esophageal squamous cell carcinoma, colorectal cancer, gallbladder cancer, and so on [20]. However, the correlation between PKM2 over-expression and OS was inconclusive in gastric cancer and pancreatic cancer [20]. As for prostate cancer, there were few study to evaluate the clinicopathological features of PKM2, and these papers reported the correlation between high gleason score and PKM2 expression as we showed [21, 22].

Accumulating evidence has suggested that PKM2 is more than a regulator of metabolic reprogramming, suggestive of multiple non-metabolic functions during carcinogenesis. Recent studies has suggested that PKM2 translocate into the nucleus and acts as a transcriptional co-activator of β-catenin and hypoxia-inducible factor 1α (HIF-1α), cooperating to control cell proliferation and glucose catabolism, respectively [23, 24]. Yang et al demonstrated inhibition of PKM2 by PKM2 shRNA reduced cell proliferation by EGFR-promoted β-catenin transactivation [23]. Other studies reported PKM2 dimer is an active protein kinase, while the tetramer is an active pyruvate kinase [25]. PKM2 activators, such as TEPP-46 and DASA-58 has been suggested to promote tetramer formation and to result in suppressing tumorigenesis [14]. As for the relation of PI3K/Akt/mTOR pathway and PKM2, Sun et al elucidated PI3K/Akt/mTOR pathway up-regulates PKM2 via HIF-1α. They reported combination therapy of Rapamycin (mTOR inhibitor) and 3-BrPA (glycolytic inhibitor) showed significant efficacy on mouse embryonic fibroblasts cells [26]. Of course, to validate the result, we need to experiment other PTEN null CRPC cell line, to elucidate whether PKM2 is a dimer or a tetramer in C4-2AT6 under hypoxia and to evaluate further clinical tissues.

As with our previous reports, C4-2AT6 had higher expression of pAKT and higher sensitivity to PI3K/AKT/mTOR inhibitor [15]. In our study we elucidate that C4-2AT6 under hypoxia shows resistance to PI3K/Akt/mTOR inhibitor. Consequently, we noted PKM2 was up-regulated by inhibition of PI3K/Akt/mTOR pathway, and the control of the expression of PKM2 showed significant efficacy for C4-2AT6 under hypoxia. These results indicate targeting PKM2 is one of promising therapies for CRPC.

## MATERIALS AND METHODS

### Reagents

Rabbit monoclonal antibody for phospho-Akt (Ser473), S6 ribosomal protein (S6), phospho-S6 ribosomal protein, phospho-4E-BP1, cleaved PARP and PKM2 were obtained from Cell Signaling Technology^®^, Rabbit polyclonal antibody for cleaved caspase-3 was obtained from Cell Signaling Technology^®,^ mouse monoclonal antibody for HIF1-a was purchased from Santa Cruz Biotechnology^®^ and mouse monoclonal antibody for β-actin was purchased from Sigma^®^. WST reagents were obtained from Takara Bio (Kyoto, Japan). NVP-BEZ235 and RAD001 were kindly provided by Novartis (Basel, Switzerland) without compensation.

### Cell lines and culture

The C4-2 prostate cancer cell line was obtained from UroCorInc. (Oklahoma City, OK). The C4-2AT6 cells were grown in RPMI-1640 (InvitrogenTM) containing 10% charcoal-stripped fetal bovine serum (C-FBS), as previously reported [27, 28]. C4-2 cells were grown in RPMI-1640 containing 10% charcoal-stripped fetal bovine serum, at 37°C in a humidified 5% CO_2_ atmosphere. These cells were passaged upon reaching confluence during a 6 month period. We named this cell line C4-2AT6 (Androgen-ablated treatment for 6 months). C4-2 cells were routinely maintained in RPMI-1640 supplemented with 10% fetal bovine serum at 37°C in a humidified atmosphere with 5% CO2. To evaluate hypoxic condition, we used multi gas incubator, and C4-2AT6 cells were maintained in a humidified atmosphere with 5% O2.

C4-2 and C4-2AT6 cells have a characteristics to PTEN null, androgen receptor positive. We previously reported phosphorylated Akt (pAkt) was significantly up-regulated in C4-2AT6 cells compared to C4-2 cells and the administration of docetaxel induced further up-regulation of pAkt in C4-2AT6 cells. Furthermore, C4-2AT6 cells showed significantly higher resistance to docetaxel than C4-2 cells *in vivo* as well as *in vitro* [15].

The PKM2 plasmids were stably transfected into C4-2AT6 cells using the Xtreme GENE HP DNA Transfection Reagent (Roche, San Francisco, CA, USA) according to the manufacturer's instructions. The empty pBApo-CMV Neo vector (Takara Bio Inc, Shiga, Japan) was also transfected into C4-2AT6 as control vector.

### WST cell viability assay

C4-2 and C4-2AT6 cells were seeded onto 96-well plates, allowed to attach for 24 hours, and then treated with different concentrations of RAD001. At the end of the incubation period, QST reagents were added to each well and the cells incubated for 1 hour. Cell viability was estimated by colorimetry by reading the color intensity in a plate reader at 570 nm.

### Cell extracts and western blot analysis

Whole cell extracts were obtained using RIPA buffer composed of 50 mM tris-HCL (pH 7.5), 150mM NaCl, 1% NP-40, 0.5% deoxycholate, and 0.1% sodium dodecyl sulfate, and containing protease inhibitors. In Western blotting, 50 μg of total protein was separated by sodium dodecyl sulfate-polyacrylamide gel electrophoresis on 12.5% acrylamide gel and then transferred to a nitrocellulose membrane. The blots were incubated with peroxidase-labeled secondary antibody (Dako). Signals were detected using enhanced chemiluminescence reagents with the ECL Plus™ Western Blotting Detection System and analyzed. Intensity was quantified using an LAS 3000 system (Fujifilm, Tokyo, Japan).

### Small interfering RNA

PKM2 expression was transiently down-regulated using the following predesigned duplex siRNA (Applied Biosystems). The sense sequences of the siRNAs were as follows: si-*PKM2*, 5’-CUUGUCCGAUGUUACCCAAtt-3. Cells were transiently transfected with 20 nmol of the respective siRNAs using Lipofectamine 2000 (Life Technologies, Carlsbad, CA). After 12 hours, siRNA was removed by replacing the culture medium with fresh RPMI 1640 containing 10% FBS, and the cells were further incubated for 36 hours. A mock-transfected control was prepared using the transfection reagent only.

### Patient selection

Samples of archival paraffin-embedded tissue sections and clinicopathological features were obtained from 156 patients with prostate cancer diagnosed and operated on at Keio University hospital. One hundred and sixty-seven patients underwent curative surgery that included radical prostatectomy for localized prostate cancer between January 2005 and December 2008. None of the patients had received hormonal treatment before the operation. After radical prostatectomy patients were followed by serum PSA level. PSA relapse was defined by an elevation of serum PSA over 0.2 ng/ml. Histology of the specimens was evaluated by two independent pathologists using haematoxylin-eosin stained tissue preparations.

### Tissue samples and immunohistochemistry

All the tissue samples were fixed in 10% formalin, embedded in paraffin and cut into 4- μm-thick sections. All pathological specimens were reviewed again by genitourinary pathologists to unify the reproducibility of the diagnosis. As for the pathologic stage, all neoplasms were classified according to the 2006 TNM staging system. This retrospective clinical study was approved by the ethics committee of Keio University Hospital (Approval number 20160084). We carried out immunohistochemical staining for PKM2. Tissue sections were deparaffinised in xylene, and hydrated by immersion in graded alcohols and finally in distilled water. After antigen retrieval with citric acid (pH 6.0), endogenous peroxidase activity was blocked with 1% hydrogen peroxide. The tissue sections were then incubated with a blocking solution of 5% dry milk in PBS. Primary antibody (monoclonal PKM2 antibody at a dilution of 1:200 Cell Signaling Technology^®^) was applied, and they were incubated with secondary antibodies conjugated to peroxidase-labeled dextran polymer. Visualization of the immunoreaction was performed with diaminobenzidine (DAB) and counterstaining was conducted with 10% hematoxylin.

Two authors independently evaluated immuno-reactivity. They were blinded to the clinical course of the patients. The two investigators gave 0-2 points to each samples as the intensity of expression and the total score greater or equal to 3 was assign to “high intensity group” and the others was to “low intensity group”.

### Statistics

The association between each clinincopathological parameter and PKM2 expression of tumor were validated using chi-square test or Mann-Whitney *U*-test. Biochemical recurrence- free survival was estimated using the Kaplan-Meier method and was compared by using the log-rank test. A p value <0.05 was considered significant.

## SUPPLEMENTARY MATERIALS FIGURES


