# Supplementary material for: PKM2 under hypoxic environment causes resistance to mTOR inhibitor in human castration resistant prostate cancer
Source: Oncotarget. 2018 Jun 12;9(45):27698–707. doi: 10.18632/oncotarget.25498 (PMC6021245; doi:10.18632/oncotarget.25498)
Supplement: Supplementary file 1 [file oncotarget-09-27698-s001.pdf]

PKM2 under hypoxic environment causes resistance to mTOR inhibitor in human castration resistant prostate cancer

SUPPLEMENTARY MATERIALS

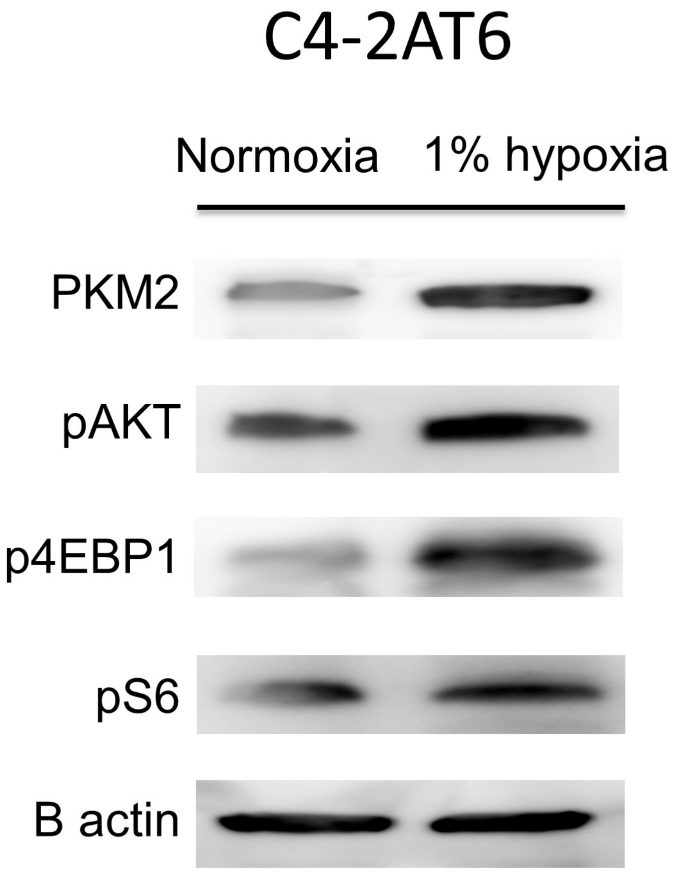

Supplementary Figure 1: 1% hypoxia also up-regulates the expression of PKM2 as well as PI3K/Akt/mTOR pathway. Expression of PKM2 as well as pAkt, pS6 and p4EBP1 was up-regulated by exposure to 1% O2.

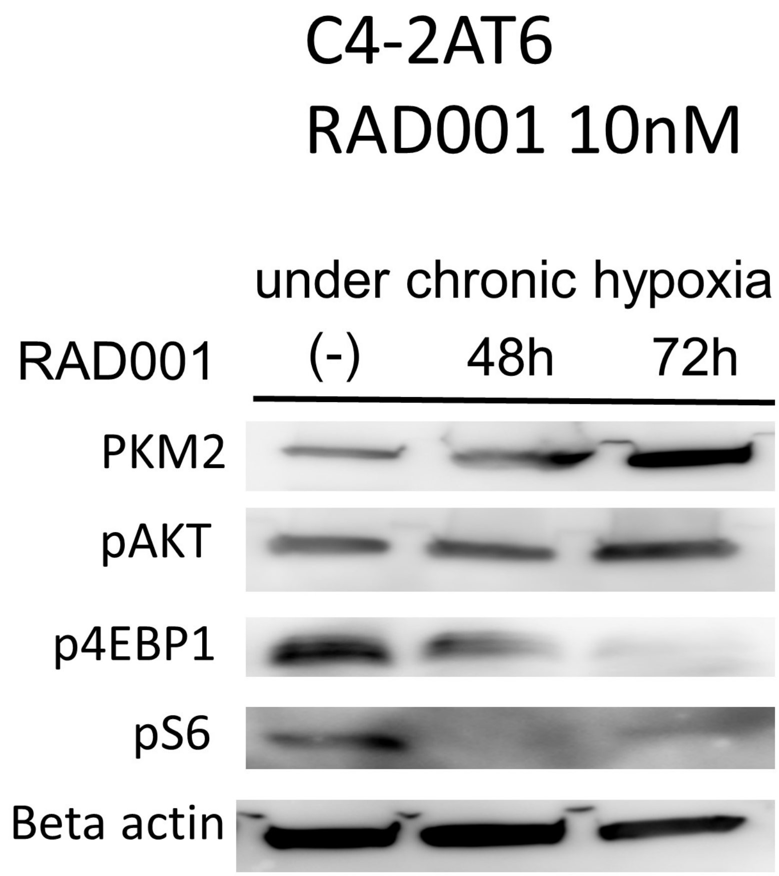

Supplementary Figure 2: Long term treatment with low dose RAD001 also up-regulates the expression of PKM2. 10nM RAD001 inhibited the expression of p4EBP1 and pS6 72 hr after administration in C4-2AT6 cells under hypoxia. Expression of PKM2 significantly increased in a time-dependent manner**.**

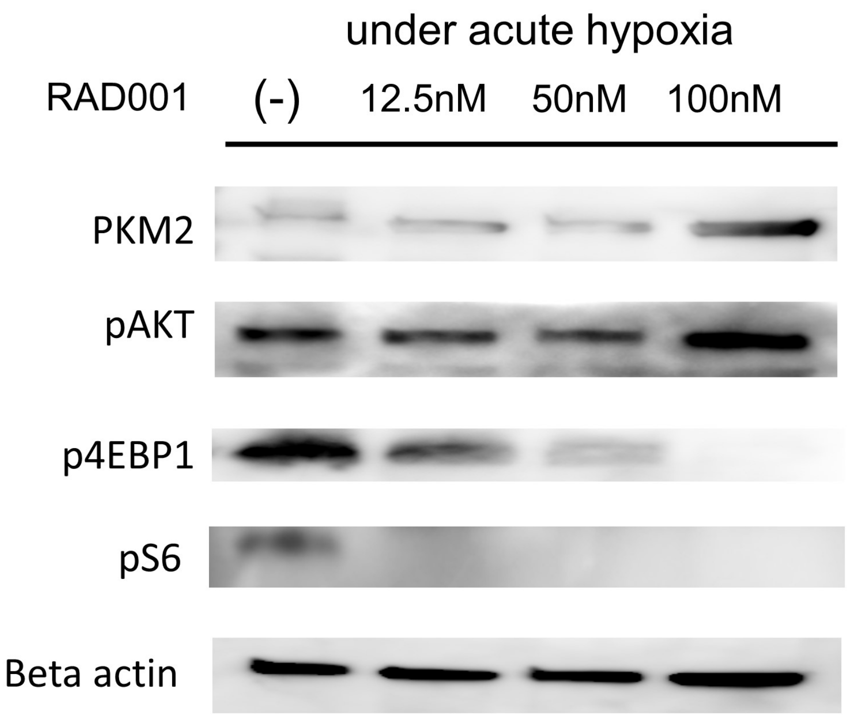

Supplementary Figure 3: The induction of PKM2 by 48h RAD001 under acute hypoxia also has dose-dependence. C4-2AT6 cells were exposed for 24 hr to the concentrations of RAD001 indicated under acute hypoxia. pS6 and p4EBP1 were inhibited by 50nM RAD001 and 100nM RAD001, respectively. The expression of PKM2 was up-regulated under 100nM RAD001.

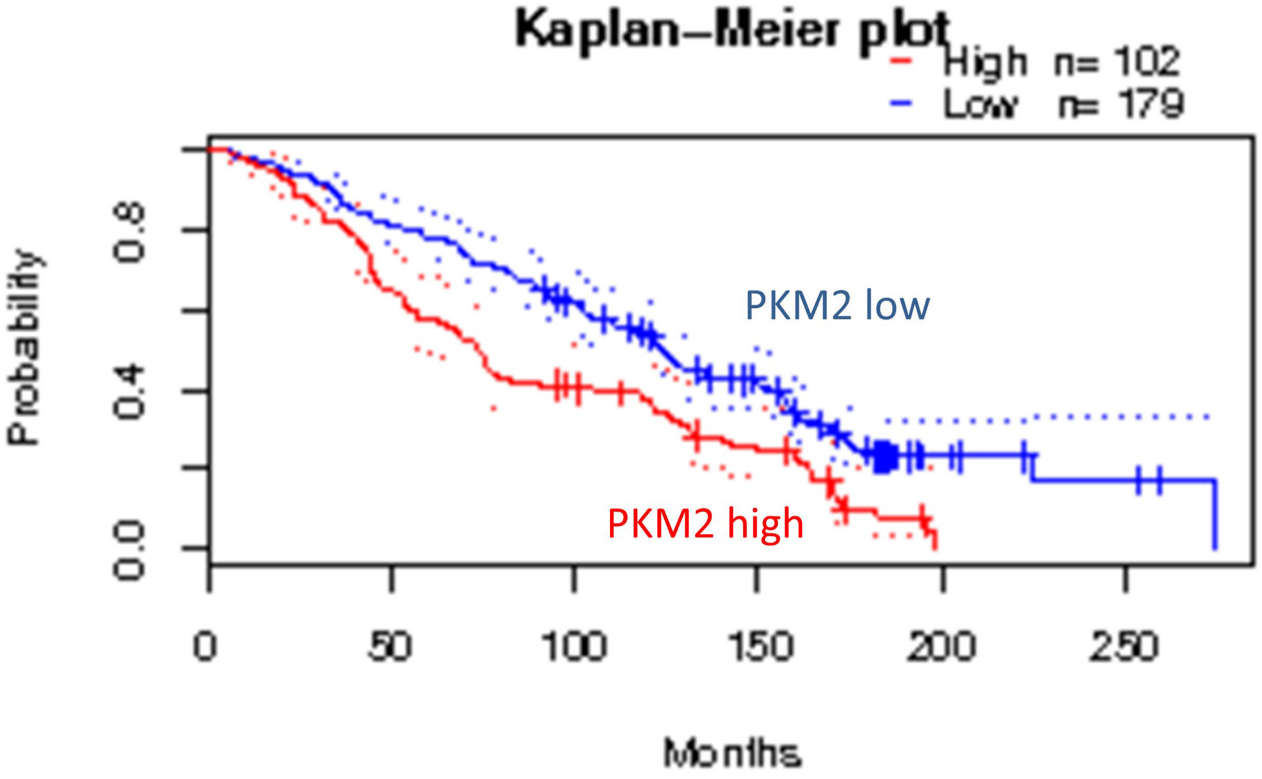

Supplementary Figure 4: Bioinformatics analyses on the microarray dataset show that high expression of PKM2 is correlated with overall survival in prostate cancer. In the microarray dataset from the GEO database (GSE16560), high expression of PKM2 was associated with significant decreases in overall survival in prostate cancer ($p<0.001$).
